# Patient adherence to out-patient psychiatric care for neurotic and affective disorders (Should I stay, or should I go?)

**DOI:** 10.1192/j.eurpsy.2022.1584

**Published:** 2022-09-01

**Authors:** A. Soboļevs, K. Kozlova, E. Tērauds, L. Sīle

**Affiliations:** 1 Rīga Stradiņš University, Faculty Of Medicine, Riga, Latvia; 2 Riga Centre of Psychiatry and Addiction disorder, Psychiatry And Addiction Medicine, Riga, Latvia

**Keywords:** out-patient, affective disorders, Patient adherence, neurotic disorders

## Abstract

**Introduction:**

Referral is not a necessity for a patient who wants to get psychiatrist consultation in Latvia. The good thing about it is the availability and the possibility to consult with highest educated mental health specialist for any person in society without barriers. On the other hand, there is an overwhelming work load for psychiatrists.

**Objectives:**

To explore the prevalence of self-referred patients in out-patient care and the adherence to psychiatrist recommendations.

**Methods:**

The medical documentation of all consecutive first-time out-patient center “Pardaugava” psychiatrist patients over the period of 01.01.2020. to 30.04.2020. with one year follow-up was analyzed.

**Results:**

236 patients were included in the study, 31.2% of them were men. The average age was 49 (SD ± 22.65) years. Patients with Affective (F3X) and Neurotic (F4X) disorders were self-referred more often compared to Organic mental (F0X) disorder or other spectrum patients (83.3% and 77.5% vs 33.3% or 56.0%, p<0.001). Median appointment count was 4, higher in F4X (6) and lower in F3X patients (2). Majority of F4X patients (61,6%) did not follow the recommendations or stopped seeing psychiatrist, while only 13,7% were persistent. Сonversely, 48,4% of F3X patients followed the recommendations and only 43,5% stopped. In comparison, other spectrum patients followed recommendations in 32.0% of cases and ignored - in 56.6%.

**Conclusions:**

Patients were actively self-referring themselves to psychiatrist. Highest adherence to psychiatrist recommendations was found in patients with affective disorders compared to other spectrum patients. In contrast, adherence was the lowest in patients with neurotic disorders.

**Disclosure:**

No significant relationships.

